# Cell type-specific properties and environment shape tissue specificity of cancer genes

**DOI:** 10.1038/srep20707

**Published:** 2016-02-09

**Authors:** Martin H. Schaefer, Luis Serrano

**Affiliations:** 1 EMBL/CRG Systems Biology Research Unit, Centre for Genomic Regulation (CRG), The Barcelona Institute of Science and Technology, Dr. Aiguader 88, Barcelona, Spain; 2 Universitat Pompeu Fabra (UPF), Dr. Aiguader 88, Barcelona, Spain; 3 Institució Catalana de Recerca i Estudis Avançats (ICREA), Pg. Lluís Companys 23, Barcelona, Spain

## Abstract

One of the biggest mysteries in cancer research remains why mutations in certain genes cause cancer only at specific sites in the human body. The poor correlation between the expression level of a cancer gene and the tissues in which it causes malignant transformations raises the question of which factors determine the tissue-specific effects of a mutation. Here, we explore why some cancer genes are associated only with few different cancer types (i.e., are specific), while others are found mutated in a large number of different types of cancer (i.e., are general). We do so by contrasting cellular functions of specific-cancer genes with those of general ones to identify properties that determine where in the body a gene mutation is causing malignant transformations. We identified different groups of cancer genes that did not behave as expected (i.e., DNA repair genes being tissue specific, immune response genes showing a bimodal specificity function or strong association of generally expressed genes to particular cancers). Analysis of these three groups demonstrates the importance of environmental impact for understanding why certain cancer genes are only involved in the development of some cancer types but are rarely found mutated in other types of cancer.

Cancer cells accumulate genomic mutations, which give a growth advantage to these cells. To unravel the molecular mechanisms underlying cancer growth, many cancer genomes of different cancer types have been sequenced. Thereby, a large amount of frequently mutated genes has been discovered. Strikingly, the pool of mutated genes found in cancer patients differs a lot from cancer type to cancer type. This suggests that the growth advantage, which a mutation confers to the cancer cell, varies with the cell type and therefore different sets of mutations are selected in different tissues.

Some genes are associated to tumorigenesis in a broad variety of tissues (general-cancer genes) while others are mutated only in very few cancer types (specific-cancer genes) (Fig. 1A). An example of a general-cancer gene is p53, which has been found mutated in the majority of cancer types^1^. In contrast, BRCA1 is an example for a specific-cancer gene: germline loss-of-function mutations in this important double-strand DNA repair protein increase the risk by up to 80% for breast and ovarian cancers but to a much lower rate for other cancer types^2^. Significant levels of somatic mutations in BRCA1 are only found in ovarian cancer genomes^1^.

Surprisingly, cancer genes are not overexpressed in the tissues from which the tumor originate^3^. The lack of agreement between expression and pathology location suggests the presence of other factors contributing to the tissue specificity of cancer genes. Different physiological scenarios likely contribute to the tissue specificity of cancer genes^4^,^5^. First, a cancer gene might have different functions in different tissues (multifunctionality). In these cases, oncogenic transformations might be driven directly by the inactivation or constitutive over-activation of the specific function in the disease tissue. Second, cellular characteristics (e.g., sensitivity to apoptosis or rate of cell division) might affect the impact of a mutation on a cell. Finally, in unaffected tissues, another gene might compensate for the loss or activation of a cancer gene, while the compensating gene might be not expressed or inactive in the affected disease tissue (functional redundancy and compensation). In agreement with the idea that cell-intrinsic differences could explain the cell type-specificity of cancer genes, differences in the organization of signaling pathways between tissues have been shown to modulate the effect of cancer gene mutations [e.g., KRAS^6^]. However, there is evidence that also environmental interactions due to cell-extrinsic factors might modulate the effect of a mutation. For example recently, it has been described how estrogen regulates NRF2 via the PI3K–AKT pathway, which promotes the survival of BRCA1-deficient cells in estrogen-responsive tissues^7^. Another example for the impact of cellular environment on tissue specificity of cancer genes is that defective UV-induced DNA damage repair increases the risk of developing skin cancer^8^. Together, these observations suggest that an interplay between cell intrinsic properties and extracellular factors could determine the effect of a mutation in a specific tissue^4^.

The purpose of this study is to determine possible mechanisms by which the cellular environment affects the tissue specificity of cancer genes. We do so by first systematically identifying factors that determine if a gene is associated with only a few or many cancer types. Therefore, we computed a cancer gene specificity score from mutation data of 21 different cancer types (Fig. 1B). We used this score to investigate if certain protein functions are preferentially associated with specific- or general-cancer genes (Fig. 1C). We show that, besides the internal organization of the cell, exposure to cell exogenous factors (Fig. 1D) shapes the distribution of cancer gene specificity.

## Results

### Specificity of cancer genes

A recent study^1^ analyzed almost 5000 different tumor-normal pairs to detect somatic point mutations and significantly mutated genes within and across 21 tumor types. This study uncovered 224 genes with a significant enrichment of mutations in at least one tumor type and 219 genes significantly enriched in mutations in a pooled pan-cancer dataset consisting of all tumor genomes. The unified statistical framework in the Lawrence *et al.* study^1^ applied to the representative selection of 21 tumor types allowed us to identify specific (i.e., only mutated in a small number of cancer types) and general (i.e., mutated in a large fraction of cancer types) cancer genes and to investigate their functional properties in an unbiased manner.

**Figure 1:**
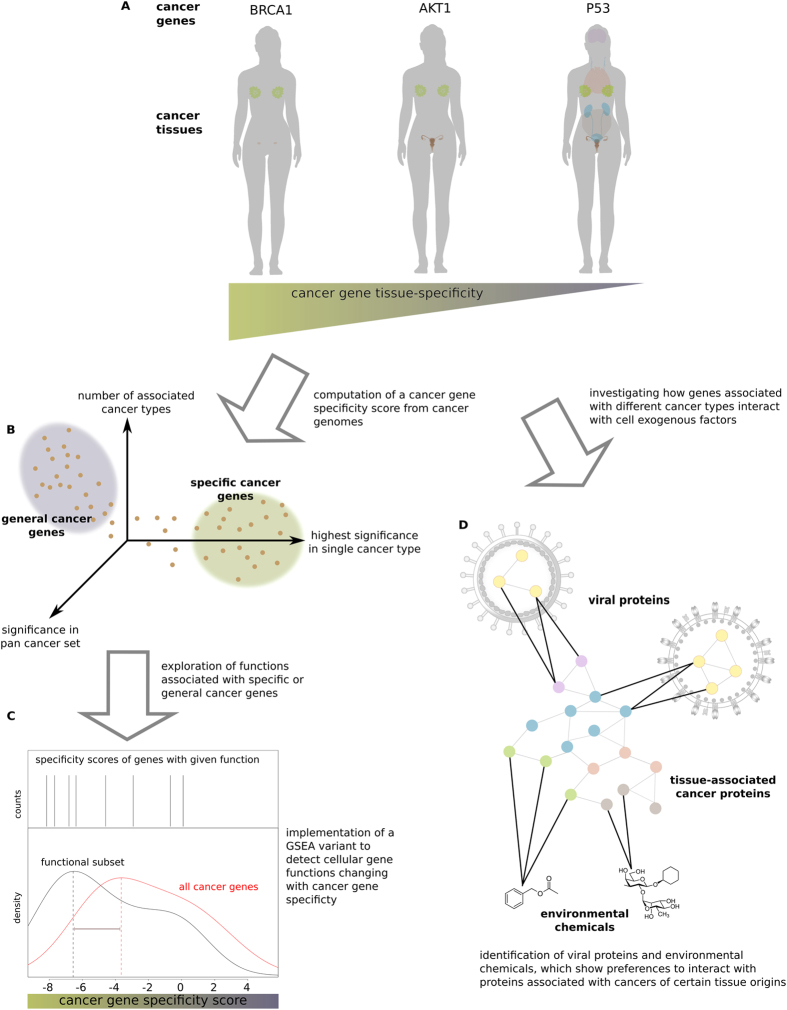
Overview of the study. (**A**) Some cancer genes (e.g. BRCA1) are only found mutated in certain types of cancers while general-cancer genes (e.g. p53) are associated with cancers from many different tissue origins. (**B**) A cancer gene specificity score was computed for each gene implementing the intuition that a high significance in a single tumor type and a low significance in the pan-cancer set are indicative of a specific-cancer gene. (**C**) The ranking of cancer genes by specificity was used to computationally detect functions that are predominantly associated with specific-cancer genes as compared to general ones (and vice versa) using a variant of the GSEA algorithm. (**D**) Proteins were grouped by the tissues in which they are involved in the development of cancer. Environmental chemicals and viral proteins frequently interacting with genes associated with one type of cancer were detected.

Following the intuition that a high significance in a single tumor type and a low significance in the pan-cancer set are indicative of a specific-cancer gene, and vice versa, that a high pan-cancer significance paired with lower significance in single tumor types is characteristic for general-cancer genes (Fig. 1B), we defined a cancer gene specificity score. This score is computed from the ratio of smallest single tumor-type mutation enrichment q value (multiple testing corrected p value) and pan cancer q value, as well as the number of tumor types in which the gene is significantly mutated (Methods; Equation (1)). This score provided us with a ranked list of all cancer genes where genes with a small score are strongly associated with a single tumor type, and genes with a high score are mutated in different tumor types (see Supplementary Table S1). We refer to this set of specificity-scored genes as the coreCancer set. The ranked nature of the coreCancer allows us to investigate if certain gene functional properties change as a function of the cancer gene specificity.

### Expression properties of general- and specific-cancer genes

As it has been previously described that cancer genes do not tend to be overexpressed in the tissues from which the cancer develops^3^, we, first, investigated the related question if general-cancer genes are more broadly expressed than specific-cancer genes to determine if ubiquitously expressed (i.e., housekeeping) proteins tend to be involved in more cancer types. Second, we examined the tissue expression patterns of highly specific-cancer genes to clarify if at least genes only associated with one type of cancer show the strongest expression in the associated tissues. For this purpose, we used a recent proteomics dataset quantifying protein expression in more than 60 healthy human tissues and cell lines^9^.

**Figure 2:**
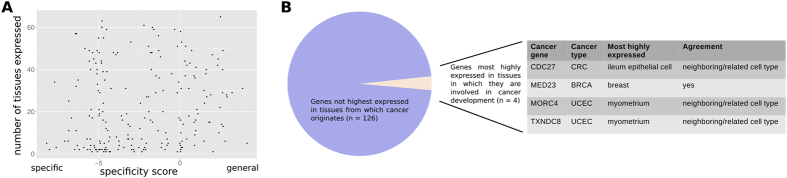
Expression promiscuity and agreement with tissue of pathology of cancer genes. (**A**) The number of tissues in which a cancer gene is expressed is shown versus its specificity. The small correlation between these variables (0.12; Pearson correlation) is not significant (p > 0.05). (**B**) We determined the tissue with the highest expression level of 130 highly specific-cancer genes (i.e., only associated with one type of cancer) and found that only 4 are expressed highest in the tissue (or in a closely related tissue) from which the respective cancer originates.

To begin with, we investigated the relation between the cancer gene specificity score and the number of tissues in which the respective protein has been detected (Fig. 2A). The correlation between these two variables was low (r = 0.12; Pearson correlation; p > 0.05; Fisher transformed Pearson’s product momentum correlation). The correlation was even lower between the raw number of tissue origins from which a gene drives cancer development and, again, the number of tissues in which it is expressed (r = 0.02; Pearson correlation). We repeated the analysis correlating the cancer gene specificity score (and the raw number of associated tissues from which the cancer originates) with the number of cell lines where the protein is detected (as given by the same proteomics resource as used before^9^). We found slightly higher correlations (r = 0.19 for the cancer gene specificity score and r = 0.06 for raw tissue number). As many of the cell lines are derived from tumors the higher correlation might indicate that genes mutated in many cancer types tend to be also expressed in many cancers.

Next, we restricted ourselves to highly specific-cancer genes, i.e. those only involved in one type of cancer. We asked if the tissue where the respective protein is most abundant tends to be the one from which cancers originate that carry mutations in this protein. We only considered solid tumors for the analysis of cancer gene expression to facilitate the association between cancer types and tissue origins and retrieved protein abundance measurements from more than 60 healthy tissues^9^. From the 130 genes associated with one solid tumor type and for which we had protein abundance information only one is most highly expressed in the tissue in which it is involved in cancer (the breast cancer-gene MED23) (Fig. 2B). Considering also the highest expression in adjacent tissues, three more genes show an agreement between expression and pathology location (the endometrial cancer genes MORC4 and TXNDC8 most highly expressed in the myometrium and colon cancer gene CDC27 most highly expressed in the ileum). The number of times we find an agreement between strongest expression and pathology location is not significant (p > 0.05; Binomial distribution). Considering RNA levels^10^ instead of protein abundance gives a similar result. Only three of the 130 genes are most highly expressed in the healthy tissues from which the cancer originates with which they are specifically associated: glioblastoma gene CHD8 is most highly expressed in brain tissues, endometrial cancer gene CUX1 in the uterus and bladder cancer gene FOXQ1 in bladder. This means that for the vast majority of cancer genes tissues exist in which the cancer gene is more strongly expressed as in the tissue where it is associated with pathology. This raises the question why it is not leading to pathology in those other tissues and which factors, if not protein abundance, determine tissue-specific pathology.

### Cellular functions associated with general- or specific-cancer genes

**Figure 3:**
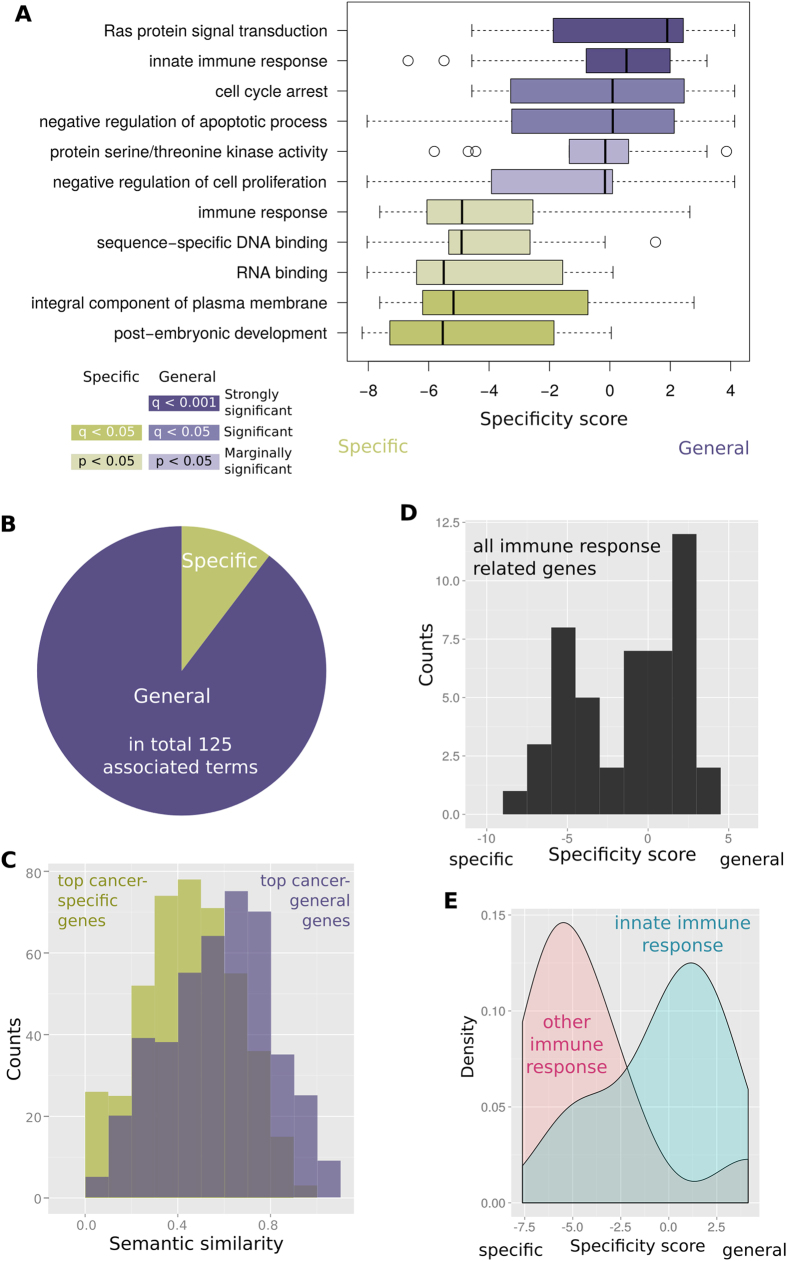
Cellular functions associated with specific- or general-cancer genes. (**A**) Enrichment of representative functional categories among specific- and general-cancer genes. Strongly associated (q < 0.05 or q < 0.001) categories are shown in darker colors, marginally significant categories (p < 0.05) in lighter colors. (**B**) Pie chart illustrating the amount of functional categories enriched among specific- (6 categories) and general- (52 categories) cancer genes. (**C**) The most cancer-general genes tend to be functionally more similar to each other (measured by the distribution of GO semantic similarities of all pairs of genes) than the most cancer-specific genes (p < 4.8e-15; Wilcoxon-Mann-Whitney test). To minimize effects due to curation differences, only genes studied 500 times or less were considered. Among those the 30 most specific and most general genes were compared with respect to semantic similarity and number of studies. The two resulting gene sets differed only with respect to functional heterogeneity and not in the number of studies (p = 0.51; Wilcoxon-Mann-Whitney test). (**D**) Immune response-related genes show a bimodal specificity value distribution. (**E**) This bimodality is caused by innate immune response genes showing the tendency to be more cancer-general and all other immune response genes to be more specific (p = 0.001; Wilcoxon-Mann-Whitney test).

To further investigate cellular functions overrepresented among specific- or general-cancer genes, we implemented a variant of the gene set enrichment analysis (GSEA), a computational method capable of detecting overrepresentation of functional classes on one end of an ordered list of genes with respect to the other end of the list (see Methods). We applied the method to the coreCancer set ordered with respect to the specificity score to detect Gene Ontology (GO) functions associated with cancer-specific and cancer-general genes. Supplementary Table S2 contains a full list of significantly enriched GO functional categories, and Fig. 3A displays the distribution of the cancer gene specificity score associated with a representative selection of the most significant terms (excluding redundant functional categories). The most highly enriched specific-cancer genes are the ones we should expect: membrane-bound, embryonic development-related, immune response, and RNA-/DNA-binding proteins (all p < 0.05; GSEA; Fig. 3A and Supplementary Table S2). There is a larger number of terms highly overrepresented among general-cancer genes (as compared to specific ones) likely reflecting their higher functional diversity (Fig. 3B). Also, as expected many of the functional categories associated with general-cancer genes represent general tumor suppression or oncogenic functions. They hint to cancer general processes crucial for different tumor types independent of the tissue origin. Examples are the suppression of apoptosis (both positive and negative regulators), interference with signaling pathways (represented for example by the GO categories “Ras protein signal transduction” and “fibroblast growth factor receptor signaling pathway” or “phosphatidylinositol-mediated signaling”), escape of cellular immune response (notably “innate immune response”), and cell cycle arrest (Fig. 3A).

The cancer genes considered here might have been studied to an uneven amount of times. As a result the observed difference in number and type of functional annotation of the top and bottom of the specificity ordered cancer gene list, could be an artifact of an elevated research attention towards cancer-general genes. To exclude this possibility, we investigated how often cancer-general genes are studied as compared to cancer-specific genes (Supplementary Fig. S1). We observed only a moderate correlation between our specificity score and the number of times a protein has been linked to articles in PubMed (r = 0.25; Pearson correlation). To exclude that this moderate, though positive correlation impacts our observations, we excluded all highly studied genes (studied more than 500 times) from the coreCancer set (Supplementary Fig. S1). In the resulting gene set, no substantial correlation between number of studies and cancer gene specificity score can be detected (r = 0.13; Pearson correlation). Repeating the GSEA on this gene set (187 instead of 224 genes), we can mostly reproduce the previously observed functional associations of specific- and general-cancer genes (Supplementary Table S3): e.g., all previous functional categories associated with specific-cancer genes remain significantly enriched (except for “post-embryonic development”). The most strongly enriched functional categories among general-cancer genes remain related to signaling (even though some drop under significance level as many of the highly studied pathway members, such as RAS genes, are removed due to the high number of times they have been studied).

We further investigated if our initial hypothesis that the higher number of functional annotations associated with cancer-specific genes is (a) caused by a higher level of functional diversity and (b) is not due to any study bias. Therefore, we compared the pairwise GO semantic similarity of all pairs of genes within the 30 most specific and within the 30 most general cancer genes (again excluding genes studied more than 500 times from the coreCancer set). We found that, indeed, general cancer genes tend to show a higher pairwise similarity as compared to specific-cancer genes (Fig. 3C). This supports our initial conclusion that the lower number of GO terms associated with specific-cancer genes is caused by the higher functional diversity of the underlying gene set.

Interestingly, functional categories related to DNA repair, whose suppression is a hallmark of most cancer types, are missing from the list of terms enriched among the general-cancer genes. Another intriguing observation is that genes involved in immune response are enriched at the top and the bottom of the specificity ordered cancer gene list (Fig. 3A). Closer inspection of all genes in the coreCancer set related to immune response revealed a bimodal distribution of cancer gene specificity scores (Fig. 3D). Intriguingly, we observed that this bimodality was caused by the strongly significant differences (p = 0.001; Wilcoxon-Mann-Whitney test) in specificity of cancer genes associated with innate immune response versus cancer genes associated with immune response but not the innate immune response (Fig. 3E). We also observed that half of the non-innate immune response genes were found mutated in lymphoma, while few of the innate immune response genes were involved in lymphoma. In contrast, the fraction of genes related to solid tumors was larger among innate immune genes (e.g., around one third involved in breast cancer).

As both DNA repair and cellular immune response are processes that cells perform in response to damage caused by mutagens or infection by pathogens, we investigated if external context effects might explain the different functional associations of specific- and general-cancer genes. Therefore, we considered cellular exposure to different mutagens, viruses and chemicals as sources of specificity in the following analyses.

### Environment-specific DNA repair pathways host tumor type-specific-cancer genes

**Figure 4:**
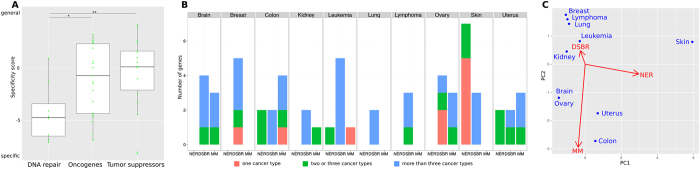
Specificity of cancer hallmark classes. (**A**) DNA repair genes (n = 8) are significantly (*p < 0.05; **p < 0.01; Wilcoxon-Mann-Whitney test) more specific than genes from the other cancer hallmark classes of oncogenes (n = 25) and tumor suppressors (n = 18). (**B**) The distribution of cancer-related DNA repair pathway genes differs between tissues. (**C**) Principal component projection of cancer tissues in DNA repair pathway space. The red arrows show the original axes in the new space.

Cancer genes can be grouped into three classes: oncogenes, tumor suppressors, and DNA repair (or stability) genes^11^. The first two classes control the balance between cell proliferation, division, and death. DNA repair proteins serve to identify and correct DNA damage and to initiate cellular response to the damage. We tested if one of these broad classes of cancer genes is more populated by specific-cancer genes than the others. We observed large differences in cancer gene specificity scores of the DNA repair genes as compared to both other groups (Fig. 4A): DNA repair genes were associated with significantly lower cancer genes specificity scores than oncogenes or tumor suppressors (p < 0.05 and p < 0.01, respectively; Wilcoxon-Mann-Whitney test).

To investigate why DNA repair proteins are associated on average with fewer cancer types than oncogenes or tumor suppressors, we extended the coreCancer gene set (which contained only 8 DNA repair genes from the set of 270 DNA repair genes annotated in GO) by adding known (somatic and germline) cancer genes from the literature (see Methods). This increased the total number of cancer genes from 224 to 670 (with 38 DNA repair genes). However, this was done at the price of the specificity ranking, as we did not have information about the strength of the association between a cancer type and a gene for most of the genes. Within this list, we associated each cancer gene with the tissues and cell types in which it had been reported to drive cancer development. We refer to this list as the extCancer (extended cancer) gene set.

We categorized DNA repair genes in the extCancer set into different DNA repair pathway classes. We considered the pathway classes nucleotide excision repair (NER), mismatch repair (MM), and double-strand break repair (DSBR), as these have a low gene overlap (Supplementary Fig. S2). This resulted in a list of 27 DNA repair genes uniquely associated with one of the three DNA repair classes. Next, we investigated how the numbers of genes involved in NER, MM, or DSBR differ across cancer genes from different tissues (Fig. 4B). This comparison revealed largely different associations across different cancer tissues, with an interesting pattern emerging (Fig. 4C):

- Skin cancer was significantly associated with the highest number of NER genes (p < 0.05; Fisher test). The high number of NER pathway genes in skin cancer makes sense, as UV light causes covalent links between adjacent pyrimidines. These dipyrimidines can be repaired by NER. However, pyrimidine transitions are frequent at unrepaired sites. Accordingly, C to T mutations are the most frequent mutation in skin cancers^12^.
- Colon has the highest number of MM genes; again, this association is significant (p < 0.05; Fisher test). Base substitution mismatches happen during DNA replication, and intestinal cells are the most rapidly dividing cells types^13^; therefore, colon cells are highly dependent on correct functioning of the mismatch repair pathway.
- Breast, leukemia, lung, and lymphoma have the highest proportion of DSBR genes with respect to the other DNA repair pathways. Double-strand breaks (DSBs) can occur under physiological and pathological conditions^14^. The physiological events happen during B and T cell development (V(D)J recombination) and in mature B cells (switch recombination). The natural occurrence of DSBs in B and T cells might explain the importance of the DSB pathways and the resulting association of leukemia and lymphoma to DSBR. Indeed, chromosomal translocations in lymphomas have been shown to be the consequence of faulty V(D)J recombination^15^. Pathological DSBs are induced by several intra- and extracellular factors (such as reactive oxygen species and ionizing radiation)^14^. For example, estrogen exposure has been identified to induce DNA DSBs^16^, by molecular mechanisms that are not fully understood. However, experimental evidence suggests that both estrogen receptor alpha (ER-alpha) activation by estradiol (the dominant estrogen)^17^ and the conversion of estrogen to genotoxic metabolites can cause DNA double-strand breaks^18^. This might cause the association between DSBR genes and breast cancer. Several studies suggest that tobacco smoke induces DSBs^19^,^20^,^21^,^22^. Therefore, DSBR-deficient lung cells might show a higher sensitivity to tobacco smoke, explaining the association between lung cancer and DSBR genes.
- Brain, kidney, ovary, and uterus show less pronounced differences in the DNA repair pathways associations.

The associations are even stronger when only specific-cancer genes are considered: all specific-cancer genes in the DSBR class are associated with estrogen-related tissues (CHEK2, breast cancer; RAD51C and RAD51D, ovary cancer; and the DSBR cancer gene BRCA1, breast and ovary). All specific-cancer genes in the MM class are associated with rapidly dividing tissue (MUTYH, colon cancer; and ABL1, leukemia; note that lymphocytes and intestinal cells are among the most rapidly dividing cell types13]. Accordingly, all five specific skin cancer genes from the NER class - DDB2, ERCC3, ERCC5, XPA, and XPC - are associated with UV-sensitive skin.

We noticed that many of the cancer genes in the NER, MM or DSBR pathway are mutated through germline variants rather than somatic mutations: 18 genes had germline mutations only, 5 somatic only and another 5 both germline and somatic mutations. The entire extCancer set contained only 66 genes with germline mutations only (from in total 670 genes) implying a 6.5-fold enrichment of genes in the NER, MM or DSBR pathway among germline mutated cancer genes. We wondered if this is caused by a general tendency of germline mutated cancer risk genes to encode DNA repair genes. Indeed, we observed a strong enrichment of DNA repair genes among germline mutated cancer genes as compared to somatically mutated (q = e-15 using the tool ConsensusPathDB^23^). We next asked if germline cancer risk genes are generally more tissue-specific as compared to somatically mutated cancer driver genes. For this purpose we compared the distributions of associated tissues between the two sets. To minimize the effect of different curation strategies on the reported number of tissues where cancer manifests we considered only the subset of 457 exclusively germline or somatic cancer genes from the cancer census database^24^. The number of associated tissues did not show any significant differences between somatic and germline cancer genes (p = 0.86; Wilcoxon-Mann-Whitney test) suggesting that none of the two cancer gene classes is more tissue-specific than the other. However, we need to note that these analyses were performed on manually curated gene lists and results might change when systematically collected data will be available.

In summary, these observations suggest that the specificity of DNA repair genes is based in some cases to intrinsic cellular properties (i.e., cell division rates; generation of antibodies), while in external factors like distinct environmental mutagens, such as UV-, hormones or tobacco exposure explain their specificity.

### Viral infection determines tissue specificity of cancer genes

As demonstrated in a previous section, genes involved in the immune system show a bimodal distribution of cancer gene specificity scores in the coreCancer set (Fig. 3C): we found that the category ‘innate immune response system’ is one of the functional classes most strongly populated by general-cancer genes (Fig. 3A, q < 0.001; GSEA). In contrast, the more general category ‘immune response’ is associated with specific-cancer genes (p < 0.05; GSEA). We observed very different distributions for the cancer gene specificity scores associated to immune response genes in the innate immune response system as compared to those that are not in it (Fig. 3D; p = 0.001; Wilcoxon-Mann-Whitney test).

To understand what causes the differential specificity of innate and non-innate immune response genes (from which several though not all were annotated as being part of the “adaptive immune response”), we applied GSEA analysis to the subset of the 47 cancer genes with a function in the immune response. The strongest functional category enriched among specific-cancer genes involved in the immune response was ‘integral component of plasma membrane’ (p < 0.001; GSEA). Inspection of the analyzed gene set revealed that, among the ten most specific immune response cancer genes, five were bound to or an integral part of the plasma membrane (CD70, HLA-B, TNF, TNFRSF14, and CD79B). Four of these five proteins (CD70, HLA-B, TNF, and TNFRSF14) have described roles in antiviral adaptive response, inflammatory response to viral infection, or viral host cell entry^25^,^26^,^27^,^28^.

**Figure 5:**
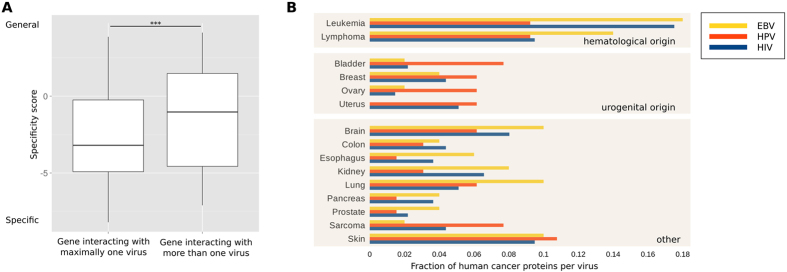
Interactions between viral and cancer proteins. (**A**) Cancer genes targeted by maximally one virus tend to be more specific (***p < 0.001). (**B**) Cancer proteins of different cancer types tend to interact with proteins from different virus types. Cancer types are grouped by similar tissue origin. Cancer proteins from hematological origin tend to interact with EBV proteins and proteins from cancer types of urogenital origin tend to interact with HPV proteins.

As viruses recognize their target cells by their membrane protein composition, we investigated if viral host cell tropism (the specificity with which viruses infect host cells) might relate to cancer gene specificity. For this, we retrieved host-virus protein-protein interaction information from VirusMentha^29^. We observed that host proteins that interact with viral proteins from maximally one virus strain were associated with significantly less tumor types than those that interact with viral proteins from several virus strains (Fig. 5A; p < 0.001; Wilcoxon-Mann-Whitney test). There was no significant difference in specificity between proteins with no viral interactions and those with interactions with only one virus strain.

For each tumor type we counted the number of proteins in the extCancer set interacting with proteins from different viruses. We considered viruses that have been implicated in carcinogenesis and for which we had sufficient PPI data: namely, Epstein-Barr virus (EBV), human papillomavirus (HPV) type 16, and human immunodeficiency virus (HIV) type 1. The infection cycle and involvement in cancer of the three investigated types of viruses is very different:

- EBV, in the herpes family, infects B cells and epithelial cells and causes several types of lymphomas and nasopharyngeal cancer^30^. EBV infection as a cause for chronic lymphocytic leukemia is under discussion^31^.
- HPV infection is associated with cervix cancer^32^ and is potentially associated with ovarian^33^, urologic^34^, and breast^35^ cancers.
- HIV infects immune system cells (T cells, macrophages, and dendritic cells) and increases cancer risk indirectly through immune suppression^36^. Cancers most strongly associated with an HIV infection are Kaposi’s sarcoma, non-Hodgkin lymphoma, and cervical cancer^37^.

Figure 5B shows how often proteins from different viruses interact with human host proteins that are associated with different types of cancer. Overall, there is good agreement between the type of cancer caused by the viruses and the fractions of cancer genes from the respective cancer types that interact with viral proteins (for clarity, we grouped related tissues together, as discussed above); for instance, more than 30% of cancer proteins that interact with EBV proteins are involved in the hematopoietic system. For both leukemia and lymphoma, the normalized number of EBV interaction partners is higher than for the other two analyzed viruses. Proteins involved in uterus cancer only interact with HPV and HIV proteins (with HPV having the higher normalized protein count). Both viruses have been implicated in cervical cancer. Interestingly, HPV also shows high protein counts for bladder, breast, and ovarian cancer as compared to the other two virus type, which supports the hypothesis of an involvement of HPV in the progression of these diseases. Finally, the normalized protein count distribution was more diverse for HIV: only colon cancer had the highest number of HIV PPI partners among the viruses. This might reflect the fact that HIV causes cancer indirectly by immune suppression.

We wondered if we would see a difference in the number of proteins with germline versus somatic mutations among the EBV, HPV and HIV viral protein interaction partners. However, we did not detect any significant differences between their numbers.

As many of the PPIs listed in VirusMentha are from small scale experiments, we aimed to confirm that the observed effect is not due to a study bias towards cancer-related proteins. Therefore, we retrieved those PPIs from an independent resource (VirHostNet^38^) that were detected in large scale studies reporting at least 500 host-virus PPIs. We excluded HIV from our analysis as the intersection between HIV interactors and cancer proteins was low (23 proteins). The resulting distribution on large scale networks (Supplementary Fig. S3) largely resembles the previously observed associations (Fig. 5): again, proteins from hematological cancer origin have the highest number of interactions with EBV proteins and proteins with urogential cancer origin tend to show higher interaction number with HPV proteins (Supplementary Fig. S3).

### Associations between environmental chemicals and specific-cancer genes

As our observations suggested that cell external factors largely determine cancer gene specificity, we studied associations between environmental chemicals and specific-cancer genes. To investigate if specific-cancer genes from different tumor types interact with different classes of chemicals, we retrieved associations between chemicals and genes from the Comparative Toxicogenomics Database (CTD)^39^. From the coreCancer list of cancer genes, we considered only genes significantly mutated in a single cancer type. We restricted our analysis to solid cancers with at least eight specific-cancer genes (bladder cancer, breast cancer, colorectal cancer, endometrial cancer, kidney clear cell carcinoma, lung adenocarcinoma, and melanoma). For each set of cancer-specific genes and each chemical, we performed a statistical test to determine if there were more associations between the chemical and the cancer genes than expected by chance. For each of the investigated cancer types, we found several environmental chemicals with a significantly high number of interactions with specific-cancer genes of one cancer type (Table 1 and Supplementary Table S4).

**Figure 6:**
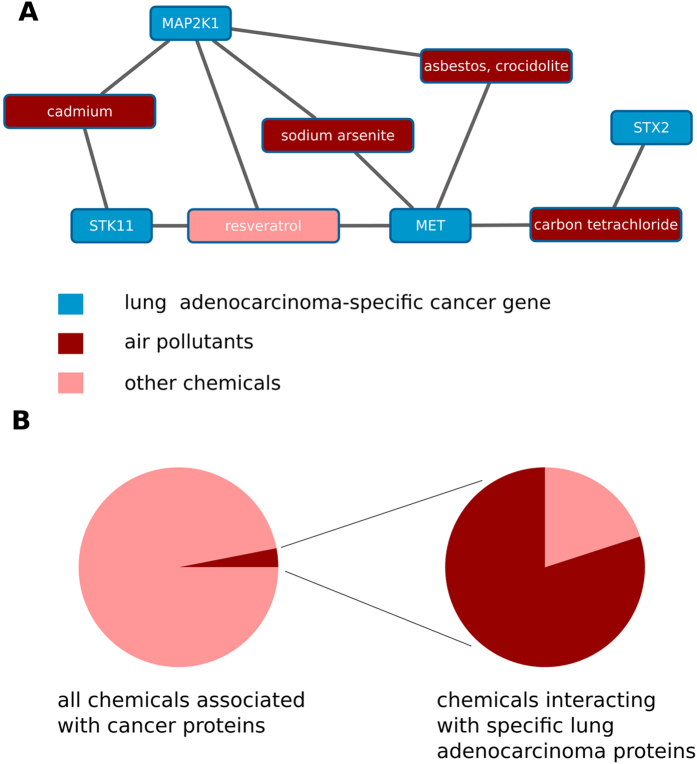
Interactions between lung cancer genes and air pollutants. (**A**) Out of the eight specific lung adenocarcinoma proteins, four proteins (blue nodes) have significant associations with environmental chemicals (red nodes). Air pollutants are shown in dark red, and other chemicals, in light red. (**B**) A high fraction of air pollutants are among the environmental chemicals that interact with specific lung adenocarcinoma genes.

In three of the seven investigated cancer types, we observed a clear association between tissues and chemicals specifically absorbed by or targeting the respective tissue origin of the cancer (see Table 1): specific breast cancer genes had a high number of interactions with hormones, specific colorectal cancer genes showed a tendency to interact with food ingredients, and specific lung cancer genes showed a large number of interactions to inhaled substances (Fig. 6A). For example, four of the five chemicals significantly associated with specific lung adenocarcinoma genes are listed as hazardous air pollutants by the United States Environmental Protection Agency (EPA). By contrasting the list of EPA air pollutants with the full set of 879 chemicals that interact with at least one specific-cancer gene (of any of the investigated tumor types), we computed a background frequency of air pollutants of 3% among specific-cancer gene-interacting chemicals (Fig. 6B). Therefore, the large percentage (80%) of air pollutants among the chemicals interacting with specific lung cancer genes is highly significant (p < 0.01, Fisher test). The chemicals significantly interacting with the other four investigated tumor types showed more heterogeneous properties and environmental origins as did the three cancer types discussed above (Supplementary Table S4).

This approach reproduced known associations between carcinogens and cancer types (e.g., asbestos is a known risk factor in the development of lung cancer^40^), but also highlighted less well-established connections between chemicals and cancer genes as potential drivers of carcinogenesis. For example, xenoestrogen bisphenol A (which is associated with specific breast cancer genes) induces higher proliferation rates^41^ and gene expression profiles characteristic for aggressive breast cancer^42^ in breast cells. However, epidemiological data on the relation between bisphenol A and breast cancer is still inconclusive^43^, and the molecular mechanisms causing the phenotypic response of breast cells undergoing bisphenol A exposure are not well understood.

To investigate if the here described associations between cancer types and environmental chemicals could be affected by biased small scale experiments, we, similar as in the previous section, repeated the analysis with only medium- and large-scale experiments. We considered only studies reporting at least 30 interactions between chemicals and genes (higher thresholds did not seem feasible as requiring at least 30 interactions already removed more than 95% of all source studies and more than 70% of all chemicals from the network). From the 18 significant associations between chemicals and cancer types (Table 1), 15 remain significant when the study size threshold is applied (Supplementary Table S5).

The strong association between genes involved in one type of cancer and chemicals specific to the environment of the tissue from which the cancer originates confirmed again the impact of environmental factors on the specificity of cancer genes.

## Discussion

It is an open question why some genes cause specifically one type of cancer but do not seem to be involved in other types. A general lack of agreement between expression location of cancer genes and location of pathology was noted a few years ago^3^, raising the question of what, if not expression, determines where a disease manifests. Even though chromatin organization has been identified as a factor biasing the mutational process towards specific genomic regions in a cell type-specific manner^44^ and tissue-specific exposure to environmental mutagens causes cancer type-specific mutational profiles^45^,^46^, there have been no conclusive answers to the question why some genes get frequently mutated in cancers from one tissue origin but not in other cancer types.

With the advent of large scale sequencing studies of cancer genomes from different cancer types, it has become evident that many genes are only found mutated in a small number of cancer types^1^,^46^. Here, we quantify for the first time the specificity of associations between genes and cancer types and perform a systematic study of the properties of genes at both ends of the specificity-ordered list of cancer genes.

On an anecdotal level, there is evidence that cell type-specific differences in signaling cause cell type-specific differences in disease manifestation. An example is APC, which shows a strong association with colon cancer, which can be explained by its fine tuning of Wnt-dependent proliferation in rapidly dividing colon crypt cells^47^. The idea of context-specific functions of cancer genes, which determines their cancer type specificity, is supported by systems level analyses demonstrating that disease genes tend to participate in tissue-specific processes^48^. Context-dependent functions of disease genes would explain why the effect of a mutation might differ among tissues independently of their expression levels: associations to processes crucial for pathology in one tissue and not in others would trigger malignant transformation upon mutation specifically in the respective tissue.

Here, we systematically quantify functional differences between cancer genes associated with few different cancer types versus cancer genes involved in many different types of cancer. In contrast to the aforementioned cell type-specific functional differences as a contributor to cell type-specific pathology, we identify the environment of the cell as an important factor that determines the effect of a mutation on cancer development. Conversely, we find signaling related functions strongly enriched among general-cancer genes.

Most importantly, we identified DNA repair in response to cell type- and environment-specific factors as an important contributor to cancer gene specificity. This might explain why cancer genes are often expressed at low to average levels in the tissues in which they drive cancer development: DNA repair genes usually have a low basal expression level, and their expression only increases after DNA damage occurs^49^.

The relation between DNA repair and tissue-specific diseases has been noted before. For example, it has been shown that genetic defects in the NER pathway lead to Xeroderma pigmentosum, a hereditary skin malignancy often accompanied by skin cancer, due to accumulation of UV-induced mutations^8^. Also, it has been proposed that differences in DNA repair between tissues contribute to the tissue specificity of trinucleotide disorders^50^. The high rates of intestinal tumors resulting from germline MM mutations have been explained with the high proliferative rates of intestinal epithelial cells^51^. Recently, it has been hypothesized that DNA repair might generally contribute to the tissue specificity of cancer genes^52^.

Interestingly, most of the DNA repair genes with relevance in cancer are germline mutated. The high number of DNA repair genes among cancer risk genes has been noted previously^24^,^53^.

Another source of specificity that we identified are host-virus protein-protein interactions. Viral tropism depends mostly on the host factor’s membrane receptor composition, as they are hijacked by the virus to function as entry gates into the cell for the virus (e.g., the high specificity with which HIV infects CD4 + T cells is due to the specific interaction between the viral envelope protein gp120 and the surface-glycoprotein CD4 during viral entry^54^). This might explain the general enrichment of membrane-bound receptors among the specific-cancer genes that we observed. Another more general interpretation of the high number of receptors among specific-cancer genes follows from a recent study^55^ in which we observed that receptors often function as cell type-specific mediators of signaling, while other functional classes (such as kinases) have more general roles. Therefore, it is not surprising that we see them associated with a higher number of cancer types.

Our finding that host-virus PPIs might affect cancer gene specificity is in agreement with the concept of modularity of disease networks (as illustrated with a number of examples from the literature in^56^) and in particular the observation that virus interacting proteins tend to be in network proximity of disease proteins^57^.

As our observations were hinting towards a strong impact of environmental factors on the tissue specificity of cancer proteins, we directly tested for an enrichment of interactions between environmental chemicals specifically absorbed by a tissue and proteins specifically associated with cancer in the same tissue. We observed very strong associations between chemicals from different classes and cancer types. For example, among environmental chemicals most highly enriched in interactions with lung cancer-associated proteins, we found more than 25-fold as many air pollutants as expected by chance. This analysis recovered known mutagens but also provides a valuable resource highlighting yet unknown relations between environmental pollutants and cancer development.

Data sets aggregated from high numbers of small scale experiments are notoriously biased towards proteins of high research interest (such as cancer proteins). This can lead to erroneous conclusions when not taken into account^58^. Here, we explicitly test whether our observations on associations between cancer genes in different tissues, environmental factors and cellular processes are affected by this type of study bias. We can show that our observations are not merely an artifact of stronger research interest in cancer-general genes or studies only testing associations between known carcinogens and cancer genes.

In our analyses, we demonstrate the importance of environmental impact for understanding why certain cancer genes are only involved in the development of some cancer types but are rarely found mutated in other types of cancer. The lack of success to explain cancer gene specificity from cell endogenous properties alone (such as relating expression levels to location of pathology) has been puzzling researchers for decades^59^.

We restricted the analyses described here to drivers of cancer undergoing non-silent mutations as the large number of sequenced exomes used in this study provided us with the means to define a high confidence set of cancer drivers. However, the extent to which other alteration types play a role in cancer progression becomes increasingly clear: differential expression of numerous genes in cancer cells have been observed for decades, copy number alterations and epigenetic changes have been systematically cataloged in recent years^60^. Even the importance of silent mutations has been demonstrated for many cases^61^. Future analyses, similar to the one presented here, could identify cancer type-specificity of other alteration types and factors driving them. To this end, a recent study identified protein complexes differentially expressed in a cancer type-specific manner^62^.

Recent research^63^ suggests that even genetic interactions between mutations (such as co-occurence and mutual exclusivity) change in a cancer type-specific manner. This means that different cancer types not just select for the presence of different sets of mutations but also for the frequencies with which combinations of them are found in the same patient. The reasons for the tissue-specificity of genetic interactions are still to be explored. However, this plasticity will likely also contribute to the cancer type-specific distribution of disease mutations.

The ongoing cancer genomes sequencing projects will allow to apply the here described method to define cancer-specific genes on an increasing scale. At the same time, efforts to map gene-chemical and host-virus interaction networks for a multitude of environmental pathogens will expand our knowledge on interactions between environmental factors and human genes. A future challenge will be to use network approaches to take the step from identifying general mechanisms that govern cancer gene specificity to predicting causal relations between specific pathogens or chemicals and carcinogenesis. This might reveal the involvement of previously considered harmless factors in disease even without the consultation of epidemiological data.

The here described contributors to cancer gene specificity illustrate how important it is to incorporate both cell endogenous and exogenous information to understand why a single mutation could have different effects on different cell types. It remains to be tested how much the cellular environment also contributes to the intra-cancer type mutational heterogeneity and to which degree differential exposure to pathogens explain why two patients with the same cancer type can differ so substantially in the mutated gene set.

## Methods

### Cancer gene sets

The analyses in this manuscript were done on two cancer gene sets: the specificity-scored coreCancer set and the larger extCancer set. To construct the coreCancer, we retrieved^1^ the smallest single-tumor type mutation enrichment q value (mostQ), the pan-cancer q value (panQ), and the number of tumor types in which the gene is significantly mutated (# tumor types). For each of the 224 cancer genes a specificity score was computed:


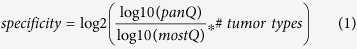


The extCancer gene set was compiled by merging coreCancer with all cancer genes from the cancer gene census (downloaded 08/27/2014)^24^ and from a recently published census of germline cancer genes^53^. Cancer genes were manually associated with the tissue origin from which it drives cancer development using the provided annotations in the different datasets. Clinically diverse cancer types were joined if they originated from the same tissue (e.g., the annotations for melanoma, skin basal cell, and skin squamous cell were joined).

### Detection of functions enriched among specific- or general-cancer genes

A variant of the GSEA algorithm^64^ was implemented in which the median of the specificity score in the functional category under consideration was computed as a gene set statistic. To assess the significance, the assignment of scores to genes was randomized 1000 times and computed the fraction of randomized gene set statistic larger (specific p value) or smaller (general p value) than the observed gene set statistic. The resulting p values for multiple testing were corrected using the Benjamini-Hochberg method, and GSEA was applied to all GO classes containing more than eight cancer genes.

### Gene annotations

Genes were functionally classified based on uniprot keywords (downloaded 09/08/14)^65^ or GO annotations (downloaded 08/12/14). For the GSEA function enrichment analysis only GO terms were considered. To study the specificity of hallmark classes, we retrieved protein associations from uniprot considering the keywords ‘proto-oncogene’, ‘tumor suppressor’ and ‘DNA repair’. For the classification into different DNA repair pathways we assigned genes to the GO classes ‘GO:0006289 nucleotide-excision repair’, ‘GO:0006298 mismatch repair’, and ‘GO:0006302 double-strand break repair’.

Semantic similarity of gene pairs was computed as the mean of the similarity of all GO terms (as given by a information content based measure^66^) implemented in the R GOSim package^67^.

As a proxy for the number of times a protein has been studied, we retrieved the NCBI curated links from genes to studies (provided by the PubMed FTP server; downloaded on 01/08/15)^68^.

### Interactions between environmental chemicals and genes

All interactions between chemicals and genes were retrieved from CTD^39^. We considered both direct physical binding and indirect associations, such as the regulatory impact of a chemical on gene expression. These associations reflect the various ways exposure to chemicals can impact protein levels and function. To ensure high quality and biological relevance of the associations, interactions based on only one evidence were removed. Approved cancer drugs were not considered for further analyses and are removed from Table 1.

## Additional Information

**How to cite this article**: Schaefer, M. H. and Serrano, L. Cell type-specific properties and environment shape tissue specificity of cancer genes. *Sci. Rep.* **6**, 20707; doi: 10.1038/srep20707 (2016).

## Supplementary Material

Supplementary Information

Supplementary Dataset 1

Supplementary Dataset 2

Supplementary Dataset 3

## Figures and Tables

**Table 1:** Associations between environmental chemicals and specific-cancer genes.

	chemical	#interactions	p value	tissue-specific exposure
BRCA	bisphenol a	6 (43%)	0.00000	Hormone-like properties (estrogenicity)
	estradiol	6 (43%)	0.00013	Estrogen
	resveratrol	4 (29%)	0.00000	Selective estrogen receptor modulator *
	choline	3 (21%)	0.00000	Estrogen induces endogenous synthesis of choline **
	quercetin	3 (21%)	0.00000	Quercetin exacerbates estrogen-induced breast tumors in rats ***
	arsenic trioxide	3 (21%)	0.00002	Arsenic trioxide represses estrogen receptor expression
CRC	resveratrol	4 (33%)	0.00000	naturally occurring in many spermatophytes like vines and peanuts
	glucose	3 (25%)	0.00000	monosaccharide found in plants, absorbed into bloodstream during digestion
	copper sulfate	3 (25%)	0.00000	used for food fortification ****
	folic acid	3 (25%)	0.00000	B vitamin naturally occurring in many foods and used as a food supplement
	carbon tetrachloride	3 (25%)	0.00000	Has been used for fumigation of grains until the 80 s
	oxygen	3 (25%)	0.00000	Chemical element (naturally occurring in all types of foods)
	coumestrol	3 (25%)	0.00057	Natural organic compound found variety of foods such as soybeans and sprouts
LUAD	resveratrol	3 (38%)	0.00000	
	asbestos, crocidolite	2 (25%)	0.00000	EPA hazardous air pollutant
	cadmium	2 (25%)	0.00000	EPA hazardous air pollutant
	carbon tetrachloride	2 (25%)	0.00005	EPA hazardous air pollutant
	sodium arsenite	2 (25%)	0.00074	EPA hazardous air pollutant

We identified chemicals interacting more often than one would expect by chance (p < 0.01; χ2 test) with groups of genes associated with a single cancer type (BRCA – breast cancer, CRC – colorectal carcinoma, LUAD – lung adenocarcinoma). Only chemicals are shown that are associated with at least 20% of the specific cancer genes of the respective cancer type. p values are multiple testing corrected. Evidence of tissue-specific exposure is provided *^69^^, **^^70^^, ***^^71^^, ****^^72^.
