# Supplementary material for: Cell type-specific properties and environment shape tissue specificity of cancer genes
Source: Sci Rep. 2016 Feb 9;6:20707. doi: 10.1038/srep20707 (PMC4746590; doi:10.1038/srep20707)
Supplement: Supplementary Information [file srep20707-s1.pdf]

Cell type-specific properties and environment shape tissue specificity of cancer genes

Martin H. Schaefer[1,2,*], Luis Serrano[1,2,3]

Author affiliations:

[1]EMBL/CRG Systems Biology Research Unit, Centre for Genomic Regulation (CRG), Dr. Aiguader 88, Barcelona, Spain

[2]Universitat Pompeu Fabra (UPF), Dr. Aiguader 88, Barcelona, Spain

[3]Institució Catalana de Recerca i Estudis Avançats (ICREA), Pg. Lluís Companys 23, Barcelona, Spain

*Corresponding author: martin.schaefer@crg.eu

Supplementary Figure S1. Relation between cancer gene specificity and number of studies. The number of recorded links between genes and PubMed articles is plotted against the cancer gene specificity score for (A) all genes and (B) genes with fewer than 500 links to articles. Pearson correlation values are indicated.

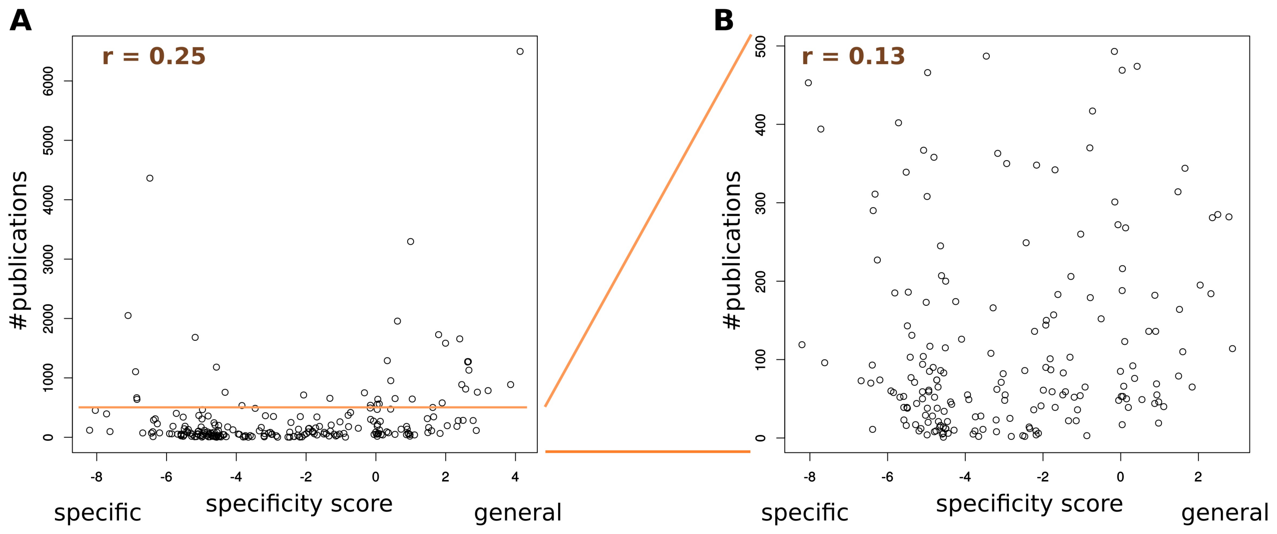

Supplementary Figure S2. Genes in DNA repair pathway subclasses. Number and overlap of genes in the three considered DNA repair pathway classes are shown: nucleotide excision repair (NER), mismatch repair (MM), and double-strand break repair (DSBR).

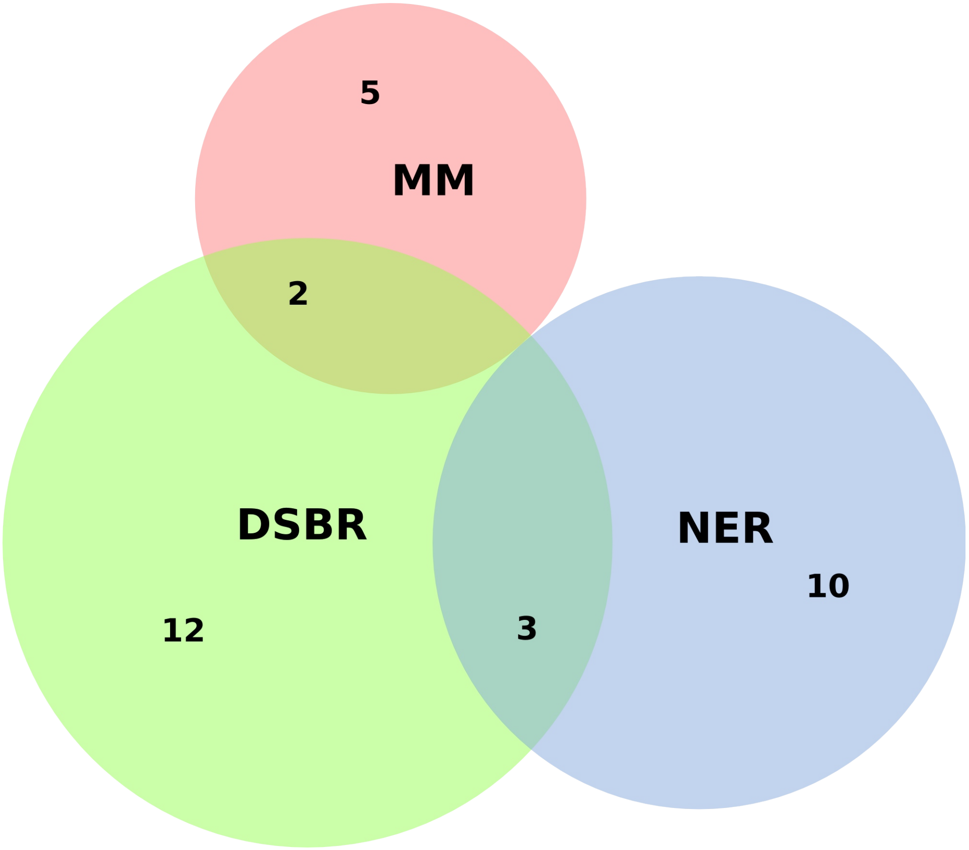

Supplementary Figure S3. Interactions between viral and cancer proteins in large-scale studies. Normalized protein counts are shown of cancer proteins interacting with EBV and HPV proteins. Only interactions from large-scale studies from the VirHostNet were considered.

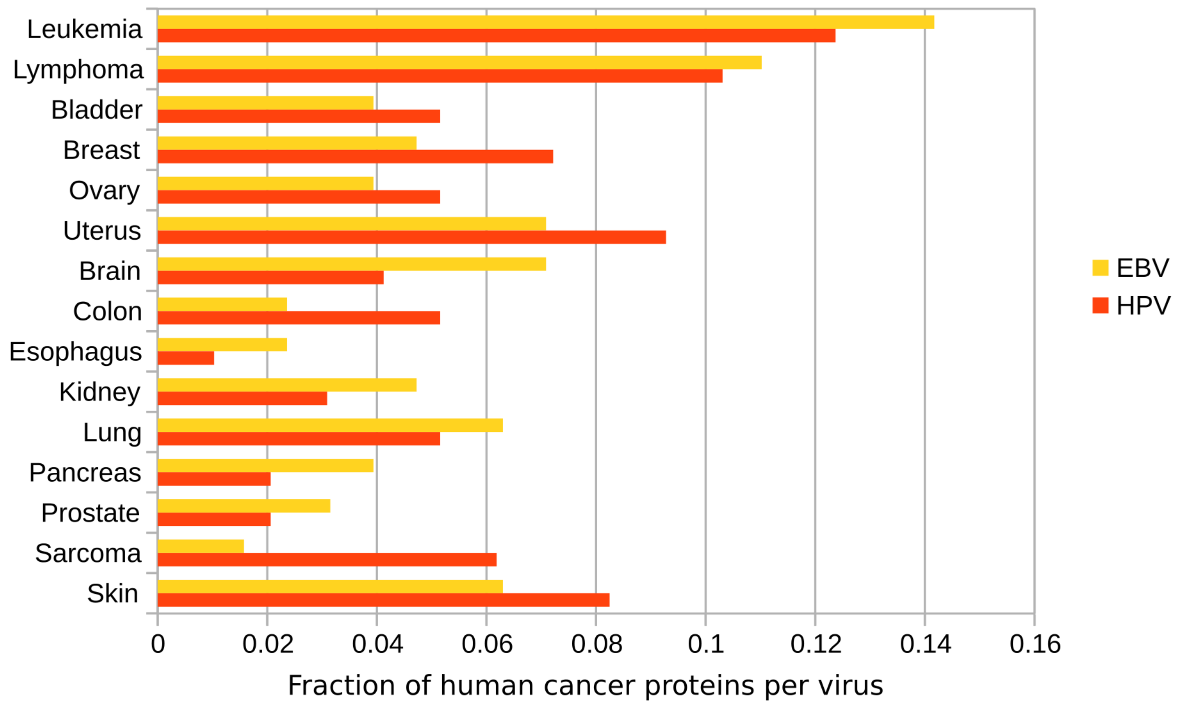

Supplementary Table S3. GO terms most significantly enriched among specific- or general-cancer genes before and after removing highly studied genes.

general/specific	name	id	previous p	p after removing highly studied genes
specific	integral component of plasma membrane	GO:0005887	3.00E-004	0.01
	RNA binding	GO:0003723	8.00E-004	0.004
	post-embryonic development	GO:0009791	0.0023	0.18
	sequence-specific DNA binding	GO:0043565	0.0051	0.008
	external side of plasma membrane	GO:0009897	0.023	0.045
	immune response	GO:0006955	0.0423	0.002
general	MAPK cascade	GO:0000165	3.00E-004	0.08
	neurotrophin TRK receptor signaling pathway	GO:0048011	1.00E-004	0.02
	protein binding	GO:0005515	1.00E-004	0.089
	epidermal growth factor receptor signaling pathway	GO:0007173	0	0.034
	innate immune response	GO:0045087	0	0.005
	Fc-epsilon receptor signaling pathway	GO:0038095	0	0.024
	fibroblast growth factor receptor signaling pathway	GO:0008543	0	0.05
	Ras protein signal transduction	GO:0007265	0	0.06
	negative regulation of neuron apoptotic process	GO:0043524	0	0

Supplementary Table S5. Environmental chemicals interacting with specific breast cancer (BRCA), colorectal carcinoma (CRC), lung adenocarcinoma (LUAD) genes in medium- and large-scale studies.

	chemical	n original	p original	n large scale	p large scale	comment
BRCA	bisphenol a	6 (43%)	0.00000	5	8.73E-006	
	estradiol	6 (43%)	0.00013	4	0.007683225	
	resveratrol	4 (29%)	0.00000	1	0.092539751	
	choline	3 (21%)	0.00000	3	3.32E-010	
	quercetin	3 (21%)	0.00000	2	8.28E-008	
	arsenic trioxide	3 (21%)	0.00002	0	-	
CRC	resveratrol	4 (33%)	0.00000	3	6.04E-011	
	glucose	3 (25%)	0.00000	1	2.21E-006	
	copper sulfate	3 (25%)	0.00000	3	1.74E-011	
	folic acid	3 (25%)	0.00000	2	1.53E-005	
	carbon tetrachloride	3 (25%)	0.00000	0	-	
	oxygen	3 (25%)	0.00000	2	4.58E-006	
	coumestrol	3 (25%)	0.00057	3	3.19E-004	
LUAD	resveratrol	3 (38%)	0.00000	2	9.21E-008	
	asbestos, crocidolite	2 (25%)	0.00000	0	-	
	cadmium	2 (25%)	0.00000	1	0.018158317	(cadmium chloride)
	carbon tetrachloride	2 (25%)	0.00005	1	0.001530229	
	sodium arsenite	2 (25%)	0.00074	1	0.013427697	
